# Third-line antiretroviral therapy in Africa: effectiveness in a Southern African retrospective cohort study

**DOI:** 10.1186/s12981-015-0081-8

**Published:** 2015-12-01

**Authors:** Graeme Meintjes, Liezl Dunn, Marla Coetsee, Michael Hislop, Rory Leisegang, Leon Regensberg, Gary Maartens

**Affiliations:** Clinical Infectious Diseases Research Initiative (CIDRI), Institute of Infectious Disease and Molecular Medicine (IDM), Faculty of Health Sciences, University of Cape Town, Anzio Road, Observatory, Cape Town, 7925 South Africa; Division of Infectious Diseases and HIV Medicine, Department of Medicine, University of Cape Town, Cape Town, South Africa; Department of Medicine, Imperial College London, London, UK; Aid for AIDS Management, Afrocentric Health Pty Ltd, The Boulevard, Block G, Searle Street, Woodstock, 7925 South Africa; Division of Clinical Pharmacology, Department of Medicine, University of Cape Town, Groote Schuur Hospital Old Main Building, Observatory, Cape Town, 7925 South Africa

**Keywords:** Virologic failure, Human immunodeficiency virus, HIV, Antiretroviral therapy, Salvage, Third-line

## Abstract

**Background:**

An increasing number of patients in Africa are experiencing virologic failure on second-line antiretroviral therapy (ART) and those who develop resistance to protease inhibitors (PI) will require third-line ART, but no data on the outcomes of third-line are available from the region. We assessed the virologic outcomes and survival of patients started on salvage ART in a Southern African private sector disease management programme.

**Methods:**

Retrospective observational cohort study with linkage to the national death register. Adults (≥18 years) who started salvage ART between July 2007 and December 2011 were included. Salvage ART was defined by inclusion of darunavir or tipranavir in an ART regimen after having failed another PI. For Kaplan–Meier (KM) analysis, patients were followed up until event, or censored at death (only for virologic outcomes), leaving the programme, or April 2014.

**Results:**

152 patients were included. Subtype was known for 113 patients: 111 (98 %) were infected with subtype C. All 152 had a genotype resistance test demonstrating major PI resistance mutations. Salvage drugs included were: darunavir/ritonavir (n = 149), tipranavir/ritonavir (n = 3), raltegravir (n = 58), and etravirine (n = 8). Median follow-up was 2.5 years (IQR = 1.5–3.3). 82.9 % achieved a viral load ≤400 copies/ml and 71.1 % ≤50 copies/ml. By the end of the study 17 (11.2 %) of the patients had died. The KM estimate of cumulative survival was 87.2 % at 2000 days.

**Conclusions:**

Virologic suppression was comparable to that demonstrated in clinical trials and observational studies of salvage ART drugs conducted in other regions. Few deaths occurred during short term follow-up. Third-line regimens for patients with multidrug resistant subtype C HIV in Africa are virologically and clinically effective.

## Background

The emergence of resistance to antiretroviral drugs is an inevitable consequence of expanding access to antiretroviral therapy (ART) and longer durations of exposure. Currently less than 15 % of patients in ART programmes in Africa are on second-line ritonavir-boosted protease inhibitor (PI) regimens [1, 2], but this proportion will increase significantly over time. Recent studies have shown that most patients failing second-line ART in resource-limited settings do not have major PI resistance-associated mutations, implying that failure is due to poor adherence rather than the development of resistance [3, 4]. However, with more patients failing on second-line ART regimens for longer durations this will likely change [5]. The threat of “untreatable” multi-drug resistant HIV after second-line failure in Africa was recently highlighted [6].

The 2013 World Health Organization (WHO) ART guidelines recommended that national programmes should develop policies and conduct pilot studies for third-line ART for patients with multi-class resistance after second-line failure [7]. Newer ART salvage regimens, which include drugs such as darunavir, raltegravir and etravirine, have been associated with reasonable virologic suppression rates in clinical trials [8–10], but clinical outcome data are limited. Variation in drug resistance patterns and susceptibility have been reported in relation to HIV subtype differences [11–13], but no data exists on effectiveness of salvage regimens in Africa where the majority of patients are infected with subtype C.

We evaluated virologic and clinical outcomes on salvage ART in the Southern African private health care sector amongst patients who had previously experienced virologic failure on a PI-containing regimen and in whom a genotype antiretroviral resistance test (GART) prior to starting salvage ART demonstrated at least one major PI mutation. Our broader aim was to inform advocacy and planning for third-line ART in programmes in sub-Saharan Africa.

## Results

On 31 December 2011 there were a total of 96,527 adult patients (≥18 years) receiving ART in the Aid for AIDS (AfA) programme, 152 of whom fulfilled our inclusion criteria for salvage therapy. The characteristics of our cohort are shown in Table 1. One-hundred and forty patients reported being exposed to an NNRTI or had this recorded on the AfA database. Eight patients gave no history of NNRTI exposure and there was no record of NNRTI exposure on the AfA database (two of whom had NNRTI resistance mutations). In four patients NNRTI exposure history was not available because they joined the AfA programme only after they had been treated with ART outside of the programme and history of their original regimens was not available on the database; in two of these patients NNRTI resistance mutations were detected. All patients had failed a PI regimen (97 % failed a ritonavir-boosted PI regimen). A total of 185 GARTs were performed prior to salvage therapy: 123 patients had one, 25 had 2 and 4 had 3 GARTs. The subtype was recorded for 113 patients: 111/113 (98 %) were subtype C.

**Table 1:** Demographic and clinical characteristics and genotype antiretroviral resistance test profile of the 152 patients when starting salvage ART

Characteristic	Number (%) or median (IQR)
Demographic	
Female gender	75 (49 %)
Age (years)	44 (39.8–48)
Clinical	
CD4 count when initially started ART (cells/mm^3^) (n = 109)	102 (31–186)
HIV VL when initially started ART (copies/ml) (n = 100)	176,893 (73,425–500,000)
CD4 count when started salvage ART (cells/mm^3^) (n = 152)	153 (41–329)
HIV VL when started salvage ART (copies/ml) (n = 152)	82,831 (20,060–233,778)
Number of ART regimens prior to salvage ART	3 (3–4)
Known to have originally initiated ART with dual NRTI regimen	53 (34.9 %)
Duration of ART exposure prior to salvage ART (years) (n = 122)^a^	8.9 (6.9–10.4)
Duration of PI exposure prior to salvage ART (years) (n = 148)^b^	5.1 (3.4–7.2)
Genotype antiretroviral resistance test (GART)^c^	
Thymidine analogue mutations^d, e^	
0	45 (30 %)
1–2	35 (23 %)
≥3	72 (47 %)
Major protease inhibitor mutations^f^	
1–2	38 (25 %)
≥3	114 (75 %)
Stanford resistance scores	
Lopinavir	70 (40–90)
Darunavir	10 (0–20)
Etravirine^g^	5 (0–15)
Lopinavir score > 29 (intermediate/high level resistance)	136 (89.5 %)
Lopinavir score > 59 (high level resistance)	88 (57.9 %)
Darunavir score > 29 (intermediate/high level resistance)	14 (9.2 %)
Darunavir score > 59 (high level resistance)	0 (0.0 %)
Etravirine score > 29 (intermediate/high level resistance)	28 (18.4 %)
Etravirine score > 59 (high level resistance)	7 (4.6 %)

147 were treated in South Africa, 4 in Swaziland and 1 in Namibia

*ART* antiretroviral therapy, *IQR* interquartile range; *NRTI* nucleoside reverse transcriptase inhibitor, *PI* protease inhibitor, *VL* viral load

^a^In 30 insufficient data on ART history was available to calculate ART duration

^b^In 4 insufficient data on PI history was available to calculate ART duration

^c^If a patient had more than one GART, then resistance mutations detected in any GART were included in these analyses

^d^Thymidine analogue mutations were defined as: M41L, D67N, K70R, L210W, T215Y/F and K219Q/E

^e^Other major nucleoside reverse transcriptase inhibitor mutations present were: M184V (n = 84), K65R (n = 7), L74V (n = 11), Q151M (n = 4) and T69 insertion (n = 1)

^f^Major protease inhibitor mutations were defined as: D30N, V32I, M46I/L, I47V/A, G48V/M/A/S/T/Q/L, I50L/V, I54V/T/A/S/L/M, L76V, V82A/C/T/S/F/L/M, I84V/A/C, N88S/T/G and L90M

^g^Few patients were taking non-nucleoside reverse transcriptase inhibitor drugs at the time the pre-salvage GART was performed

The following salvage drugs were prescribed: darunavir/ritonavir (n = 149), tipranavir/ritonavir (n = 3), raltegravir (n = 58) and etravirine (n = 8). NRTI drugs were included in 149 salvage regimens: tenofovir (TDF)/emtricitabine (FTC) in 105, TDF/FTC/zidovudine (AZT) in 22, AZT/lamivudine (3TC) in 9, and other NRTI combinations in 13. In 2 patients although salvage therapy was authorised it could not be verified from documentation available that they had been dispensed salvage therapy. These 2 patients were, however, included in the analyses as we could not determine that they definitely had not been dispensed salvage therapy.

**Fig. 1:**
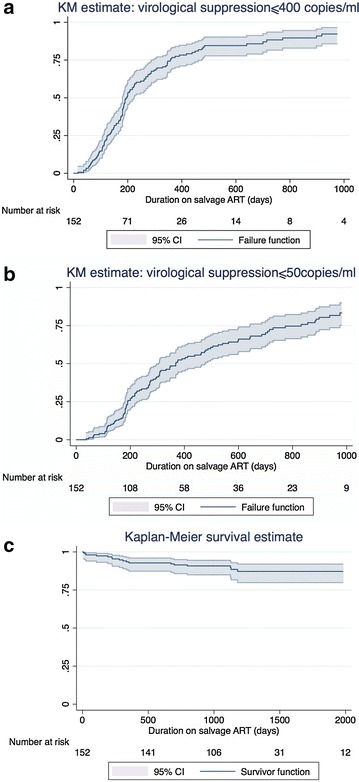
Kaplan–Meier curves showing time to virologic suppression or death. **a**, **b** Show Kaplan–Meier estimates of time to suppression of HIV viral load ≤400 and 50 copies/ml respectively. All 152 patients were included in these analyses and patients were censored at death, loss to the programme or on 30 April 2014. The median time to suppression ≤400 copies/ml was 194 days and to ≤50 copies/ml was 368 days. Viral load measurements were generally performed 6 monthly, partly explaining the delayed suppression; **c** shows Kaplan–Meier estimates of time to death analysis. Patients were censored at 30 April 2014 for this analysis (vital status was known for all patients on this date)

The median duration of follow-up on salvage therapy was 2.5 years (IQR = 1.5–3.3). By date of administrative censor (30 April 2014), 17 of 152 patients (11.2 %) had died. 145/152 (95.4 %) patients had at least 1 viral load done on salvage therapy: 126/145 achieved a VL ≤400 copies/ml (86.9 %) and 108/145 achieved a VL ≤50 copies/ml (74.5 %). Of all 152 patients, 82.9 % were therefore documented to have suppressed VL ≤400 copies/ml and 71.1 % VL ≤50 copies/ml. Kaplan–Meier (KM) estimates of cumulative proportions of all 152 patients suppressing to a VL ≤400 copies/ml at 1000 days was 92.2 % and VL ≤50 copies/ml was 83.4 % (Fig. 1a, b). Factors associated with VL suppression ≤50 copies/ml are shown in Table 2. In multivariate analysis, we found women were more likely to suppress (aOR = 2.45, 95 % CI = 1.07–5.63), whereas patients with higher VL and higher levels of darunavir resistance were less likely to suppress (aOR = 0.38, 95 % CI = 0.21–0.70 per log_10_ increase in VL, and aOR = 0.59, 95 % CI = 0.39–0.90 per 10 point increase on Stanford database, respectively). There was a non-significant trend towards the inclusion of raltegravir in the salvage regimen being associated with a higher odds of suppression (aOR = 2.38, 95 % CI = 0.95–5.95).

**Table 2:** Multivariate logistic regression analysis of factors associated with HIV viral load suppression ≤50 copies/ml on salvage ART^a^

Variable	Unadjusted odds ratio (95 % CI)	p value	Adjusted odds ratio (95 % CI)	p value
Age (per year increase)	1.00 (0.95–1.05)	0.96	1.00 (0.94–1.06)	0.98
Female gender	2.42 (1.16–5.01)	0.02	2.45 (1.07–5.63)	0.04
HIV viral load at start of salvage ART (per log10 unit increase)	0.30 (0.17–0.53)	<0.001	0.38 (0.21–0.70)	0.002
Darunavir resistance score (per 10 point increase on Stanford database)	0.54 (0.38–0.76)	<0.001	0.59 (0.39–0.90)	0.01
Raltegravir in salvage regimen	1.11 (0.54–2.30)	0.77	2.38 (0.95–5.95)	0.06

^a^All 152 patients are included in this analysis. Those who did not have a viral load performed or who died before having a suppressed viral load recorded were regarded as having failed to suppress

**Fig. 2:**
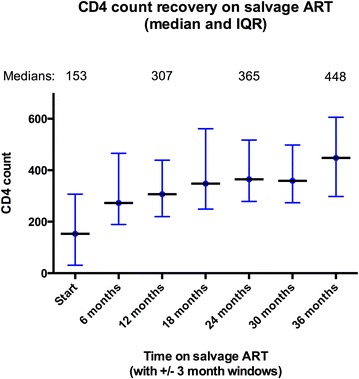
CD4 count prior to start of salvage ART and at 6 months intervals on salvage ART. The *graph* shows the median and interquartile range for CD4 counts (for those patients who had CD4 count performed) prior to starting ART salvage, then at 6 months intervals on salvage ART (with a ±3 month window for each 6 month interval)

The cumulative incidence of virologic rebound after suppression was 19 and 13.9 % of those suppressing to VL ≤400 and ≤50 copies/ml respectively. The median CD4 count increased from 153 to 448 cells/mm^3^ by 3 years on salvage ART (Fig. 2). The KM estimate of cumulative survival was 87.2 % at 2000 days (Fig. 1c). One patient stopped ritonavir of their own accord due to gastro-intestinal side effects. There were no other interruptions or substitutions for intolerance or toxicity.

## Discussion

A high proportion of these Southern African private sector patients on ART salvage therapy for a median of 2.5 years achieved virologic suppression. Factors independently associated with virologic suppression were female sex, lower darunavir resistance score, lower VL at start of salvage ART, and use of raltegravir (although the latter just failed to achieve statistical significance). The KM estimate of survival at 2000 days was 87 %. These results are striking given that this cohort of patients had previously failed multiple lines of therapy.

The proportion of patients achieving virologic suppression in our study was near to that achieved in the ANRS 139 TRIO trial conducted in France in which patients with multiclass resistant HIV were treated with darunavir, raltegravir, etravirine and either NRTIs or enfuvirtide. In that single-arm trial, 88 % of participants achieved virologic suppression (VL <50 copies/ml) at 96 weeks [14]. While a clinical trial may represent a select patient group, several observational studies of patients with multiclass resistant HIV treated with darunavir-based regimens have also reported virologic suppression (VL <50 copies/ml by week 48) being achieved in over 50 % of patients. This includes studies conducted in other developing countries: Brazil (83 % suppression [15] and 73 % suppression [16]) and Mexico (69 % suppression [17]). The inclusion of raltegravir in such darunavir-based regimens has been associated with improved virologic outcomes [16, 18].

The need for evidence regarding the implementation of third-line ART in resource-limited settings has been recognized by the WHO [7]. Our data demonstrated the effectiveness of third-line ART in a programme where the predominant subtype was C, in contrast to clinical trials of salvage drugs done in settings where non-C subtypes predominate. Our findings are in line with those of prior studies, which suggested that raltegravir and darunavir regimens had equivalent efficacy in patients with B and non-B subtype infections and that resistance to these drugs was not more prevalent with non-B subtypes [19–23]. The fact that the majority of patients achieved virologic suppression on regimens including darunavir/ritonavir, raltegravir and NRTIs suggested this could be a standardized third-line regimen, with individual adjustments based on GART.

Important limitations of our study were the retrospective observational study design, which could have resulted in selection and information bias, and potential limited generalizability to the public sector. While there are important socioeconomic differences between private and public sector patients in the region, which could affect outcomes, we felt that our results are applicable to public sector programmes as similar treatment guidelines were followed and 5 year outcomes were previously reported to be similar between AfA and a large South African public sector programme [24]. Of note is that the South African public sector ART programme has recently approved third-line therapy using similar selection criteria to AfA’s. A key strength of our study is that we reported on clinical outcomes.

## Conclusions

AfA has been providing ART, GART and new salvage drug options for longer than public sector programmes, thus providing us with the first opportunity to assess outcomes on salvage drugs in an African setting. These data provide the first evidence of the potential effectiveness of third-line regimens based largely upon a backbone of darunavir/ritonavir and raltegravir together with NRTI drugs, in a predominantly subtype C African epidemic. Availability of GART and darunavir/ritonavir and raltegravir (or dolutegravir) needs to be planned for the future as more patients fail second-line PI regimens for longer durations in public sector programmes on the continent. Our results provide evidence that third-line regimens for patients failing second-line ART with multidrug resistant HIV in Africa are likely to be effective.

## Methods

### Design

We conducted a retrospective observational cohort study of adults (≥18 years) enrolled with the Aid for AIDS (AfA) disease management programme, who started salvage ART between 1 July 2007 and 31 December 2011. Salvage ART was defined by inclusion of darunavir or tipranavir in an ART regimen after having experienced virologic failure on another PI. No patients had prior exposure to an integrase inhibitor. All patients had a GART performed prior to starting a salvage ART regimen that was used to inform choice of drugs in this regimen. These GARTs were performed at quality-assured private sector laboratories and results were sent to the patient’s doctor and to AfA. Certain laboratories reported the HIV subtype on the laboratory results sheet routinely, but for some patients (n = 39) this information was not supplied. Salvage drugs that became available during the study period were initially darunavir and later raltegravir and etravirine—these were prescribed to patients commencing salvage regimens after they became available if indicated, but were not added as single drugs for a patient already on salvage ART. Access to tipranavir was very limited.

### Setting

AfA is a private sector HIV disease management programme that provides HIV care for patients on medical insurance and corporate treatment programmes [25, 26]. HIV care includes clinical interventions coupled with clinical and patient support and on-going adherence management. Although it operates in the private sector, the AfA guidelines are similar to the WHO guidelines with a standardized approach to first-line (NNRTI-based) and second-line (protease inhibitor-based) ART regimens. Demographic and clinical data are captured in the AfA database. Salvage ART was authorized by a clinical committee after reviewing treatment history and GART. All patients starting salvage ART were contacted before being switched to assess commitment and educate them about the regimen, and thereafter monthly for adherence support until viral load was suppressed.

Important differences between AfA and the public sector ART programme in South Africa are: (1) the AfA programme started in 1998 whereas the public sector programme started in 2004; (2) in AfA some patients received initial ART with dual NRTIs for affordability reasons in the late 1990s; (3) certain drugs were available in the private sector before the public sector; (4) GART has been available for patients failing ART in AfA since 2001; and (5) darunavir was readily available to patients on the AfA programme from 2007, and raltegravir from 2009. These two drugs became available in the public sector programme in 2013.

Commencement of salvage ART was checked against the pharmacy claims history on the AfA database. Vital status was available for all patients at the date of data censor: (1) for South Africans through linkage to the South African national death register and (2) for patients outside South Africa from the clinical records. The Stanford HIV Drug Resistance Database (version 6.2.0) was used to calculate mutation scores.

### Statistical analyses

For Kaplan–Meier (KM) analyses of time to virologic suppression or death, patients were followed up until the date of the event or censored at death (only for virologic outcomes), leaving the programme, or date of administrative censor (30 April 2014). Multivariate logistic regression analysis was performed using STATA 13, StataCorp, TX, US, with all variables hypothesised to be associated with outcome included in the model. Virologic rebound was defined as ≥1 viral load (VL) >1000 copies/ml after initial suppression.
